# Risk factors for complications after adrenalectomy: results from a comprehensive national database

**DOI:** 10.1007/s00423-016-1535-8

**Published:** 2016-11-28

**Authors:** Lo Hallin Thompson, Erik Nordenström, Martin Almquist, Helene Jacobsson, Anders Bergenfelz

**Affiliations:** 1 Department of Surgery, Skåne University Hospital, 22185 Lund, Sweden; 2 Research and Development Centre Skåne, Skåne University Hospital, 22185 Lund, Sweden

**Keywords:** Adrenalectomy, Laparoscopic, Complications, Conversion

## Abstract

**Purpose:**

Most knowledge regarding outcome after adrenal surgery stems from retrospective studies reported by highly specialized centres. The aim of this study was to report a national experience of adrenalectomy with particular attention to predictive factors for postoperative complications, conversion from endoscopic to open surgery and length of hospital stay.

**Methods:**

Adrenalectomies reported in the Scandinavian Quality Register for Thyroid, Parathyroid and Adrenal Surgery (SQRTPA) 2009–2014 were included. Risk factors for complications, conversion and hospital stay >3 days were assessed using univariable and multivariable logistic regression analysis.

**Results:**

There were 659 operations. Endoscopic adrenalectomy was performed in 513 (77.8%) operations and almost half of these were robotic assisted. The median length of hospital stay was 3 (range 1–30) days. There was no 30-day mortality. In 43 (6.6%) patients, at least one complication was registered. The only factor associated with complications in multivariable analysis was conversion to open surgery odds ratio (OR) 3.61 (95% confidence interval 1.07 to 12.12). The risk for conversion was associated with tumour size OR 1.03 (1.00 to 1.06) and with malignancy on histopathology OR 8.33 (2.12 to 32.07). Length of hospital stay increased in patients with operation of bilateral tumours OR 3.13, left-sided tumours OR 1.98, hyper secretion of catecholamines OR 2.32, conversion to open surgery OR 42.05 and open surgery OR 115.18.

**Conclusions:**

The present study shows that endoscopic surgery is widely used. Complications were associated with conversion and the risk for conversion was associated with tumour size and malignant tumour. Hospital stay was short.

**Electronic supplementary material:**

The online version of this article (doi:10.1007/s00423-016-1535-8) contains supplementary material, which is available to authorized users.

## Introduction

Adrenalectomies are becoming more frequent worldwide. Contemporary imaging techniques with an increased detection of adrenal incidentalomas and new surgical techniques with reduction of morbidity and mortality with endoscopic methods [1, 2] have contributed to the increase in operations. In 1992, Gagner et al. were among the first to describe laparoscopic adrenalectomy [3]. Since then, several studies have evaluated minimally invasive surgery for adrenal lesions, which has become the standard technique for surgical treatment of adrenal tumours [4, 5]. Previous studies have compared laparoscopic with open adrenalectomy for complications, hospital stay, morbidity and mortality [2, 6, 7]. Laparoscopic adrenalectomy is widely used in both university hospitals and smaller units. Studies have evaluated risk factors for complications associated with surgical technique, surgeon volume and patient-related risk factors. Most of the studies are single-centre studies [8–10], although a few national studies that included specific hospitals or patient groups are available [11–13].

Adrenalectomies in Sweden have been registered in the Scandinavian Quality Register for Thyroid, Parathyroid and Adrenal Surgery (SQRTPA) as of 2009. All hospitals that perform adrenal surgery are participating in the registry. The aim of the present study was to evaluate a national experience of adrenal surgery over 6 years and to determine risk factors for complications, conversion from endoscopic to open surgery and prolonged hospital stay.

## Material and methods

### Register background

The SQRTPA was established 2004. In 2009, a module for adrenal surgery was added. The register is an online database and is recognized by the Swedish National Board of Health and Welfare as the national registry within the field.

Patients who underwent adrenal surgery and were reported in the registry 2009–2014 were included in the study. All procedures were performed by endocrine surgeons. Follow-up was performed at 4–6 weeks postoperatively.

### Data variables

Data were collected for age, gender, body mass index (BMI), hypertension, radiographic tumour size (largest diameter measured on adrenal-CT or MRI), side for adrenal tumour, indication for surgery, surgical technique, histopathology, conversion rate from endoscopic to open surgery, complications (bleeding with transfusion, laceration of viscus, pneumothorax, local infection/abscess and re-operation), postoperative length of stay and 30-day mortality.

Surgical techniques were grouped for the analysis. Endoscopic adrenalectomy included laparoscopic adrenalectomy (posterior and transabdominal) and robotic-assisted laparoscopic adrenalectomy (posterior and transabdominal). Open adrenalectomy included laparotomy, thoracoabdominal approach and open retroperitoneal operations. The technique used was determined by the surgeon on the basis of preoperative findings, availability of equipment and surgical experience.

The primary outcomes were risk factors affecting conversion rate, complication rate and postoperative length of stay (LOS).

The secondary outcomes were variables associated with a surgical technique, laparoscopic, versus robot-assisted adrenalectomy and endoscopic versus open adrenalectomy.

### Statistical analysis

Descriptive statistics are presented as number with valid per cent or median with minimum and maximum values. Binary logistic regression was used to analyse the association between outcome variables and predictors. Analysed outcome variables were complication (defined as bleeding with transfusion, laceration of viscus, pneumothorax, local infection or abscess and re-operation (no or yes)), re-operation (no or yes), conversion (no or yes), postoperative days before discharge (1–3 and 4–30), technique (endoscopic, open) and technique (laparoscopic, robotic). The predictors that were assessed were age, sex, BMI (less than 25 or 25 and higher), adrenal tumour side (right or left or bilateral), largest tumour size, surgical technique (laparoscopic, robotic, open), clinical syndrome of hormonal excess (none, cathecholamines, cortisol and subclinical Cushing syndrome, aldosterone), histopathology (malignant, phaeochromocytoma, other benign lesions) and conversion from endoscopic to open surgery (no or yes). Univariable analyses were conducted, and each outcome variable was assessed with each predictor. Forward multiple linear regression analyses were performed to identify models with significant predictors for each outcome variable, except for re-operation due to small numbers of events. The results from the binary logistic regressions are shown as numbers, odds ratios with 95% confidence intervals (CI) and *p* values.

All analyses were conducted using IBM SPSS Statistics 22 for Windows (IBM Corporation, Armonk, NY, USA). We considered *p* values below 0.05 as statistically significant.

## Results

Some 659 patients underwent adrenalectomy under the 6-year period in ten surgical departments. The distribution between centres is shown in Table 1. Preoperative characteristics of the cohort are summarized in Table 2.

**Table 1:** Distribution of patients between medical centres

	*n* = 659 (%)	Open	Laparoscopic	Robot
University hospital				
1	235 (35.7)	8	55	172
2	179 (27.2)	30	72	77
3	143 (21.7)	33	109	1
4	62 (9.4)	16	46	0
5	20 (3.0)	4	16	0
County hospital				
6	12 (1.8)	1	11	0
7	4 (0.6)	3	1	0
8	2 (0.3)	0	2	0
9	1 (0.15)	0	1	0
10	1 (0.15)	1	0	0

Valid percent

*n* number

**Table 2:** Results of preoperative clinical variables for the patients included in the study

		*n* = 659	Percent
Age (median, year)	59 (8–89)		
Female		372	(56.4)
BMI ≥ 25		373	(64.1)
Hypertension		215	(32.6)
Tumour side			
Left		375	(56.9)
Right		254	(38.5)
Bilateral		30	(4.6)
Tumour size (median, mm)	35 (1–200)		
Operation indication			
Clinical syndrome of hormonal excess		399	(60.5)
Suspected malignancy on radiology		215	(32.6)
Suspected malignancy on cytology		27	(4.1)
Metastasis		32	(4.9)
Size only (≥40 mm)		75	(11.4)

Valid percent. Median (min-max)

*BMI* body mass index, *n* number

The median age was 59 (range 8–89) years, and 372 (56.4%) patients were women. Some 215 (32.6%) patients had hypertension defined as blood pressure >140/90 mmHg. On radiological examination, the median size of the tumour was 35 mm (range 1–200).

Indication for operation could be one or several. The most common indication was a clinical syndrome of hormonal excess; 161 (24.4%) patients had hormonal excess of aldosterone, 113 (17.1%) patients had hypersecretion of cathecholamines, and 118 (17.9%) patients had hypercortisolism. In 215 (32.6%) patients, malignancy was suspected on the basis of radiological examination (CT or MRI). Some 32 (4.9%) patients had preoperatively diagnosed metastases and 75 (11.4%) patients underwent surgery because of a tumour size larger than 40 mm.

Perioperative characteristics are shown in Table 3. Final pathologic diagnosis was, among others, adrenal cortical adenoma in 262 (49.6%) patients and phaeochromocytoma in 100 (18.9%) patients. In total, 71 (13.3%) patients had a diagnosis of malignancy.

**Table 3:** Results of intra- and postoperative variables for the patients included in the study

		*n* = 659	Percent
Surgical technique			
Laparoscopic		263	(39.9)
Robotic		250	(37.9)
Open		146	(22.2)
Histopathology (*n* = 528)			
Malignant		71	(13.4)
Phaeochromocytoma		100	(18.9)
Other benign		357	(67.6)
Conversion		37	(5.6)
Complication			
Bleeding with transfusion		17	(2.6)
Local infection/abscess		8	(1.2)
Pneumothorax		3	(0.5)
Laceration of viscus		5	(0.8)
Re-operation		10	(1.5)
30-day mortality		0	(0)
Length of hospital stay (median, days)	3 (1–30)		

Valid percent. Median (min-max)

*n* number

Endoscopic adrenalectomy was performed in 513 (77.8%) patients and almost half of these operations were robotically assisted. In total, 146 (22.2%) patients were operated with open procedures and in 37 (5.6%) patients, conversion was performed from endoscopic to open surgery.

Some 43 (6.5%) patients had a complication and the most common was bleeding with transfusion. The 30-day mortality rate was nil. Median LOS was 3 (range 1–30) days.

### Analysis of primary outcomes

Univariable and multivariable logistic regression analysis was performed for the primary outcomes. Predictive factors for complications, conversion and LOS are presented in Tables 4, 5 and 6, respectively.

**Table 4:** Uni- and multivariable regression analysis of risk factors for complication after adrenalectomy

	Univariable logistic regression			Multivariable logistic regression		
	*n*	OR (95% CI)	*p*	*n*	OR (95% CI)	*p*
Age	658	1.02 (1.00–1.04)	0.109			
Sex						
Female	371	Ref				
Male	287	1.38 (0.75–2.53)	0.295			
BMI						
<25	209	Ref.				
≥25	372	0.73 (0.39–138)	0.336			
Tumour side			0.603			
Right	254	Ref.				
Left	374	1.39 (0.73–2.64)	0.315			
Bilateral	30	0.00 (0.00-.)	0.998			
Largest tumour size	657	1.01 (1.01–1.02)	<0.001			
Surgical technique			0.001			0.112
Laparoscopic	262	Ref.		212	Ref.	
Robotic	250	0.69 (0.30–1.56)	0.368	171	0.79 (0.33–1.92)	0.604
Open	146	2.61 (1.29–5.28)	0.007	84	2.03 (0.85–4.84)	0.109
Clinical syndrome of hormonal excess			0.157			
None	260	Ref.				
Cathecholamines	113	0.44 (0.16–1.17)	0.098			
Cortisol	117	0.42 (0.16–1.13)	0.084			
Aldosterone	161	0.62 (0.29–1.33)	0.222			
Histopathology			0.178			
Other benign	357	Ref.				
Malignant	71	1.84 (0.79–4.31)	0.157			
Phaeochromocytoma	100	0.61 (0.2–1.79)	0.364			
Conversion						
No	621	Ref.		449	Ref.	
Yes	37	2.89 (1.14–7.34)	0.026	18	3.61 (1.07–12.12)	0.038

Complications that were analysed were bleeding with transfusion, laceration of viscus, pneumothorax, local infection or abscess and re-operation analysed together

*BMI* body mass index, *OR* odds ratio, *CI* confidence interval, *Ref.* referent

**Table 5:** Uni- and multivariable logistic regression analysis of risk factors for conversion from endoscopic to open surgery

	Univariable logistic regression			Multivariable logistic regression		
	*n*	OR (95% CI)	*p* value	*n*	OR (95% CI)	*p* value
Age	659	1.01 (0.99–1.04)	0.274			
Sex						
Female	372	Ref.				
Male	287	1.11 (0.57–2.16)	0.762			
BMI						
<25	209	Ref.				
≥25	373	1.07 (0.49–2.34)	0.869			
Tumour side			0.387			
Right	254	Ref.				
Left	375	0.62 (0.31–1.23)	0.174			
Bilateral	30	0.94 (0.21–4.25)	0.932			
Largest tumour size	658	1.00 (0.99–1.01)	0.482	383	1.03 (1.00–1.06)	0.026
Surgical technique						
Laparoscopic	263	Ref.				
Robotic	250	5.14 (1.46–18.11)	0.011			
Clinical syndrome of hormonal excess			0.030			
None	260	Ref.				
Cathecholamines	113	0.38 (0.13–1.12)	0.079			
Cortisol	118	0.65 (0.27–1.56)	0.335			
Aldosterone	161	0.20 (0.06–0.66)	0.009			
Histopathology			0.208			0.005
Other benign	357	Ref.		291	Ref.	
Malignant	71	2.44 (0.9–6.66)	0.081	24	8.33 (2.12–32.07)	0.002
Phaeochromocytoma	100	1.10 (0.35–3.46)	0.87	68	0.67 (0.08–5.73)	0.718

*BMI* body mass index, *OR* odds ratio, *CI* confidence interval, *Ref.* referent

**Table 6:** Uni- and multivariable logistic regression analysis of variables associated with postoperative length of stay

	Univariable logistic regression			Multivariable logistic regression		
	*n*	OR (95% CI)	*p*	*n*	OR (95% CI)	*p*
Age	652	1.02 (1.01–1.03)	0.003			
Sex						
Female	368	Ref				
Male	284	1.13 (0.82–1.55)	0.461			
BMI						
<25	208	Ref.				
≥25	367	0.96 (0.68–1.35)	0.799			
Tumour side			0.299			0.021
Right	253	Ref.		177	Ref.	
Left	370	1.06 (00.76–1.47)	0.733	264	1.98 (1.14–3.43)	0.016
Bilateral	29	1.84 (0.85–3.99)	0.120	23	3.13 (1.08–9.06)	0.035
Largest tumour size	651	1.03 (1.03–1.04)	<0.001			
Surgical technique			<0.001			<0.001
Laparoscopic	261	Ref.		211	Ref.	
Robotic	250	1.72 (1.13–2.60)	0.011	171	1.56 (0.95–2.56)	0.081
Open	141	46.51 (23.84–90.72)	<0.001	82	115.18(32.94–402.67)	<0.001
Clinical syndrome of hormonal excess			<0.001			0.012
None	255	Ref.		162	Ref.	
Cathecholamines	113	1.03 (0.66–1.61)	0.884	84	2.32 (1.18–4.59)	0.015
Cortisol	116	1.06 (0.68–1.64)	0.805	86	1.56 (0.77–3.18)	0.219
Aldosterone	161	0.25 (0.16–0.41)	<0.001	132	0.78 (0.40–1.51)	0.458
Histopathology			<0.001			
Other benign	356	Ref.	<0.001			
Malignant	70	5.09 (2.94–8.81)	0.006			
Phaeochromocytoma	99	1.91 (1.21–3.03)				
Conversion						
No	617	Ref.		447	Ref.	
Yes	35	14.18 (4.94–40.71)	<0.001	17	42.05 (5.02–352.40)	0.001

Postoperative days before discharge, 1–3 (ref.) versus 4–30

*BMI* body mass index, *OR* odds ratio, *CI* confidence interval, *Ref.* referent

Univariable analysis showed that open surgery, large tumour size and conversion to open surgery were associated with an increased risk of complications. However, in the multivariable analysis, conversion from endoscopic to open surgery was the only independent predictive factor for postoperative complications, OR 3.61 (CI 1.07–12.12). Open surgery was also a risk factor when the surgical techniques were grouped as endoscopic versus open technique, OR 2.26 (CI 1.03–4.93), but when the operations were divided into laparoscopic, robotic and open surgery, the significance was lost (Table 4).

The risk for conversion was increased in robotic-assisted surgery, OR 5.14 (CI 1.46–18.11), in the univariable analysis. However, in the multivariable analysis, only tumour size, OR 1.03 (CI 1.00–1.06), and malignant histopathology, OR 8.33 (CI 2.12–32.07), remained predictive (Table 5).

In the multivariable analysis of LOS, tumour on the left side, OR 1.98 (CI 1.14–3.43), bilateral tumours, OR 3.13 (CI 1.08–9.06), open surgery, OR 115.18 (CI 32.94–402.67), excess of cathecholamines, OR 2.32 (CI 1.18–4.59), and conversion to open surgery, OR 42.05 (5.02–352.40), were independent predictive factors for longer hospital stay (Table 6).

### Secondary outcomes

In the univariable analysis of patient characteristics and variables depending on the technique used, older age, large tumour size, malignant histopathology and phaeochromocytoma were positively associated with open surgery when compared with endoscopic surgery. A body mass index of 25 or higher, left-sided tumours, and hypercortisolism or aldosteronism were negatively associated with open surgery.

In multivariable logistic regression analysis, larger tumour size OR 1.06 (CI 1.04–1.07) and malignant histopathology OR 11.13 (CI 4.82–25.70) were positive predictive factors for open surgery. (Suppl. Table 1 for the web).

When comparing robotic-assisted and laparoscopic adrenalectomy, older age and larger tumour size were associated with robotic-assisted adrenalectomy in the univariable analysis. In the multivariable logistic regression analysis, older age, OR 1.02 (CI 1.00–1.03), was borderline significant and larger tumour size, OR 1.02 (CI 1.01–1.03), remained the predictive factor for robotic-assisted surgery. (Suppl. Table 2 for the web).

## Discussion

In this national cohort of 659 patients undergoing adrenalectomy, some 5.1% patients had at least one complication and 1.5% required re-operation. The only predictive factor associated with complications was conversion from endoscopic to open surgery. In total, 5.6% of the procedures were converted from endoscopic to open surgery and the risk for conversion was related to larger tumour size and malignant tumours. There were no in-hospital deaths and the 30-day mortality was nil. Factors affecting LOS were left-sided tumour, bilateral tumours, open surgery, excess of cathecholamines and conversion to open surgery.

Laparoscopic adrenalectomy is the standard treatment for adrenal tumours [4]. The technique has a low rate of complications, less postoperative pain, shorter LOS and lower morbidity compared with open surgery [7, 14–16]. Laparoscopic adrenalectomy is therefore cost-effective. There has been an increase in adrenalectomy due to routine imaging and surgical techniques. Benign disease or tumours of unclear significance account for most of the increase [17]. The laparoscopic technique has evolved from transabdominal to posterior endoscopic and robotic-assisted surgery. Current studies recommend open surgery in malignant or large tumours (>100 mm) but some recent studies show equivalent results for laparoscopic surgery regarding morbidity and mortality, providing oncological principles are adhered to [18–21]. In very large or locally invasive tumours, an open surgical approach is recommended to facilitate en bloc excision of the tumour [22]. It has therefore been recommended that these patients should be treated in specialized centres in order to improve outcomes [23, 24].

There are earlier reports concerning complications in adrenalectomy. Most of them compare outcomes following laparoscopic versus open procedures and most of them are single institution studies from tertiary centres. A lack of standardized definition of complications influences the ability to compare results. Eichhorn-Warry et al. used the Clavien-Dindo classification [25] and found that laparoscopic adrenalectomy decreased the use of intensive care for complications when compared with open procedures [7]. There are only few retrospective, national investigations and none of them included all hospitals that perform adrenalectomies [6, 11–13]. It is therefore interesting that the incidence of reported complications in the literature in general is similar to what was shown in the present study [1, 10, 11, 13, 26, 27]. In the present investigation, conversion to open surgery was the only independent risk factor for complication. Although this could partly be due to bias with larger tumours being at higher risk for conversion, it could be of importance to consider when choosing surgical technique for the individual patient. For some patients, an open approach may be preferred to endoscopic adrenalectomy. The proportion of open procedures in the present report, 22.2%, is comparable with earlier studies [6, 7].

Bergamini et al., showed that age, BMI, tumour size and phaeochromocytoma are risk factors for complications but these in general were lower in referral than in non-referral centres [11]. Surgeon volume influences outcome as well and has been shown by Park et al. [28]. Furthermore, comorbidity has been associated with a risk for complications [1, 13]. These variables were not registered in the database used for the present investigation, and therefore, the possible influence of these potential risk factors could not be evaluated.

Considering the well-known effects of hypercortisolism, Cushing’s syndrome was expected as a risk factor for impaired wound healing, bleeding and postoperative infection after adrenalectomy. However, this was not shown in the present study. In contrast, Sommerey et al. found that bilateral adrenalectomy for Cushing’s disease had more severe complications [29].

The conversion rate in the present study, 5.6%, was consistent with previous reports [8, 10]. Bittner et al. collected data retrospectively from 402 patients and found that tumour size >80 mm was a predictive factor for conversion from endoscopic to open surgery [8]. This is comparable to the present results and indicates that laparoscopic adrenalectomy is used for increasingly larger lesions.

Length of hospital stay is similar in many reports and is shorter after laparoscopic than open procedures [6, 8, 9, 13]. Independent predictive factors for risk for longer hospital stay are not well explored. Of interest is that low surgeon volume and some comorbidities are associated with higher LOS [1, 28]. However, these factors were not evaluated in the present study.

Almost half of the endoscopic operations in this cohort, 37.9% in total, were robotic assisted. Robotic-assisted surgery was used in older patients and more importantly in patients with larger tumours. In the univariable analysis, there was a tendency towards a higher conversion rate compared with the standard laparoscopic technique. However, this association was lost in the multivariable analysis. Several retrospective studies on the use of robotic-assisted adrenalectomy have been published during recent years and all reported on small patient groups [30, 31]. In a meta-analysis, some advantages for robotic surgery compared with conventional laparoscopic surgery were shown such as shorter hospital stay, less intraoperative blood loss and lower risk for postoperative complications [32]. However, given the scarce data, these results should be interpreted with caution. The benefits of the robotic technique in subtotal and bilateral adrenalectomy have been discussed in a review article by Nomine-Criqui with equivocal conclusions [33].

Hospital stay is one of the largest costs in health care. Reports regarding costs associated with different surgical techniques are scarce and most have small patient groups [34, 35]. Given the higher cost for robotic surgery compared with standard laparoscopy [31, 36], it remains to be proven if the higher number of patients with large tumours that are operated with robotic surgery instead of an open approach could outweigh the increased costs for this new technique.

In the present study, patients with left-sided tumours had a longer hospital stay compared with patients operated for a tumour on the right side. However, the results are not unequivocal. One study found a longer operative time for right-sided tumours subjected to retroperitoneal endoscopic surgery [37]. Other investigations found no differences in outcome but longer operation time [38] and increased risk for surgical complications [10] in patients with left-sided tumours.

There are some limitations to the present study. The study was based on results from a database with predefined data fields, but data was analysed retrospectively. Although the register is designed for the procedure, i.e. adrenalectomy, important variables such as comorbidity, previous abdominal surgery, blood loss and operative time are missing. For non-surgical complications, for instance pneumonia, venous thrombosis and urinary tract infection, a free text field with ICD coding is available in the database. However, it cannot be ruled out that some of these complications were not coded. Finally, the database does not identify outcomes for the individual surgeon, which may affect the results.

The strength of the present study is that it involved one of the largest national series of patients operated with adrenalectomy. All hospitals that perform adrenal surgery in Sweden participate in the registry and valid data are controlled regularly by an extern audit.

## Conclusion

Within the context of these limitations, the present study shows a low risk for complications, a low conversion rate from endoscopic to open surgery and a short hospital stay for patients undergoing adrenalectomy in Sweden. The study reinforces the benefits of minimal invasive surgery for adrenal tumours on a national level.

## Electronic supplementary material

ESM 1 (DOCX 33 kb)

ESM 2 (DOCX 32 kb)
