# Supplementary material for: Risk factors for complications after adrenalectomy: results from a comprehensive national database
Source: Langenbecks Arch Surg. 2016 Nov 28;402(2):315–22. doi: 10.1007/s00423-016-1535-8 (PMC5346413; doi:10.1007/s00423-016-1535-8)
Supplement: Supplementary file 1 — (DOCX 33 kb) [file 423_2016_1535_MOESM1_ESM.docx]

Supplementary tables for the web:

Suppl. Table 1. Uni- and multivariable logistic regression analysis of variables associated with technique (endoscopic vs open surgery)

|  | Univariable logistic regression | | | Multivariable logistic regression | | |
| --- | --- | --- | --- | --- | --- | --- |
|  | n | OR (95% CI) | p | n | OR (95% CI) | p |
| Age | 659 | 1.02 (1.01-1.04) | 0.002 |  |  |  |
| Sex  Female  Male | 287  372 | Ref.  0.82 (0.56-1.19) | 0.291 | 265  202 | Ref.  0.47 (0.24-0.92) | 0.027 |
| BMI  <25  ≥25 | 209  373 | Ref.  0.66 (0.45-0.99) | 0.045 |  |  |  |
| Tumour side  Right  Left  Bilateral | 254  375  30 | Ref.  0.64 (0.44-0.93)  0.54 (0.20-1.46) | 0.048  0.019  0.222 |  |  |  |
| Largest tumour size | 658 | 1.05 (1.04-1.06) | <0.001 | 467 | 1.06 (1.04-1.07) | <0.001 |
| Clinical syndrome of hormonal excess  None  Cathecholamines  Cortisol  Aldosterone | 260  113  118  161 | Ref.  0.65 (0.39-1.07)  0.53 (0.31-0.88)  0.07 (0.03-0.17) | <0.001  0.090  0.015  <0.001 |  |  |  |
| Histopathology  Other benign  Malignant  Phaeochromocytoma | 357  71  100 | Ref.  16.74 (9.14-30.66)  3.44 (1.90-6.22) | <0.001  <0.001  <0.001 | 317  62  88 | Ref.  11.13 (4.82-25.70)  1.90 (0.89-4.07) | <0.001  <0.001  0.098 |

Surgical technique. Endoscopic (Ref.) vs open surgery

BMI; body mass index, OR; odds ratio, CI; confidence interval, Ref.; referent
