# Supplementary material for: Risk factors for complications after adrenalectomy: results from a comprehensive national database
Source: Langenbecks Arch Surg. 2016 Nov 28;402(2):315–22. doi: 10.1007/s00423-016-1535-8 (PMC5346413; doi:10.1007/s00423-016-1535-8)
Supplement: Supplementary file 2 — (DOCX 32 kb) [file 423_2016_1535_MOESM2_ESM.docx]

Suppl. Table 2. Uni- and multivariable logistic regression analysis of variables associated with technique (laparoscopic vs robotic surgery)

|  | Univariable logistic regression | | | Multivariable logistic regression | | |
| --- | --- | --- | --- | --- | --- | --- |
|  | n | OR (95% CI) | p | n | OR (95% CI) | p |
| Age | 513 | 1.02 (1.01-1.03) | 0.005 | 383 | 1.02 (1.00-1.03) | 0.055 |
| Sex  Female  Male | 284  229 | Ref.  1.11 (0.79-1.58) | 0.546 |  |  |  |
| BMI  <25  ≥25 | 153  300 | Ref.  1.12 (0.76-1.66) | 0.555 |  |  |  |
| Tumour side  Right  Left  Bilateral | 185  303  25 | Ref.  1.04 (0.72-1.50)  1.00 (0.43-2.30) | 0.971  0.819  0.992 |  |  |  |
| Largest tumour size | 513 | 1.02 (1.01-1.03) | <0.001 | 383 | 1.02 (1.01-1.03) | 0.005 |
| Clinical syndrome of hormonal excess  None  Cathecholamines  Cortisol  Aldosterone | 175  86  94  156 | Ref.  0.84 (0.50-1.41)  1.08 (0.65-1.80)  0.37 (0.24-0.58) | <0.001  0.517  0.759  <0.001 |  |  |  |
| Histopathology  Other benign  Malignant  Phaeochromocytoma | 327  28  76 | Ref.  1.29 (0.59-2.79)  1.16 (0.70-1.91) | 0.719  0.522  0.566 |  |  |  |

Surgical technique. Laparoscopic (Ref.) vs robotic surgery

BMI; body mass index, OR; odds ratio, CI; confidence interval, Ref.; referent
